# Addressing the Impacts of New Racism on Mental Health Service Use Among Culturally and Racially Marginalised (CaRM) Communities: A Q Methodology Study

**DOI:** 10.3390/nursrep16060204

**Published:** 2026-06-17

**Authors:** Eric Lim, Takeshi Hamamura, Jaya Dantas, Sender Dovchin, Stephanie Dryden, Ana Tankosić

**Affiliations:** 1 School of Nursing, Murdoch University, Murdoch, WA 6150, Australia; 2 School of Population Health, Curtin University, Bentley, WA 6102, Australia; takeshi.hamamura@curtin.edu.au (T.H.); jaya.dantas@curtin.edu.au (J.D.); 3 School of Education, Curtin University, Bentley, WA 6102, Australia; sender.dovchin@curtin.edu.au (S.D.); stephanie.dryden@curtin.edu.au (S.D.); ana.tankosic@curtin.edu.au (A.T.)

**Keywords:** Australia, culturally and linguistically diverse, culturally and racially marginalised, emotional safe space, mental health, new racism, Q methodology, nursing

## Abstract

**Background:** Culturally and Racially Marginalised (CaRM) communities in Australia encounter subtle and covert forms of prejudice, commonly referred to as “new racism”. Within healthcare settings, these experiences can shape trust, engagement, and patterns of help-seeking. Mental health nurses are often the first point of contact in care delivery, and their ability to recognise, respond to, and mitigate the impacts of new racism is critical for fostering therapeutic relationships and supporting equitable access. Understanding how CaRM communities perceive the conditions that influence their mental health service use is fundamental for informing more equitable and culturally responsive care. **Objective:** This study explored the viewpoints of CaRM community members regarding the factors they consider important for addressing new racism in healthcare systems and supporting engagement with mental health services. **Design:** Q methodology was used to identify statistically derived viewpoints that reflect shared viewpoints about the conditions perceived as critical for addressing the impacts of new racism on mental health service use. **Setting:** Participants were recruited from culturally and linguistically diverse communities across Australia through community settings, social media, and professional networks. **Participants:** Thirty-five individuals from CaRM backgrounds completed the Q-sort. **Methods:** This Q methodology consisted of five steps: (1) set up of the Q-sorting instrument, (2) selection of participants, (3) data collection, (4) factor analysis, and (5) factor interpretation. **Results:** Three distinct viewpoints were identified: (1) raising awareness of mental health issues within CaRM communities (community-focused), (2) providing visible anti-racism and culturally safe services (service-focused), and (3) recognising and formally addressing new racism within healthcare systems (policy-focused). **Conclusions:** This study offers the first empirically derived, community-informed set of viewpoints on addressing new racism in Australian mental healthcare. While exploratory, the findings highlight multi-level considerations that are potentially relevant to mental health nursing practice, and may be useful to inform future research, policy development, and service redesign aimed at strengthening cultural responsiveness and equity in mental health systems.

## 1. Background

Australia is one of the most multicultural countries in the world, shaped by its long-standing migration, humanitarian resettlement, and ongoing international mobility. According to the Australian Institute of Health and Welfare, more than seven million people in Australia were born overseas and six million people speak a language other than English [1]. In Australia, the term “Culturally and Linguistically Diverse” (CaLD) is often used to refer to communities whose race, ethnicity, skin colour, cultural practices, and spoken language differ from the mainstream Anglo-Australians [2]. In recent years, it has been highlighted that the term CaLD does not provide sufficient understanding of the experiences or impact of being othered in the mainstream community [3]. Therefore, in this paper, we use the term Culturally and Racially Marginalised (CaRM) to refer to communities that are not racialised as ‘white’ as proposed by the Diversity Council Australia [3]. CaRM communities in Australia encompass a diverse range of populations, including temporary migrants such as international students or temporary skilled workers, refugees and asylum seekers who usually settle through Humanitarian Support Programmes, and first-generation permanent residents and citizens of migrant background [4].

People belonging to CaRM communities are associated with a higher risk of experiencing poorer mental health compared to the mainstream community [5]. It is well-recognised that CaRM populations are faced with acculturation and acculturative stressors arising from socio-cultural differences from their country of origin, linguistic discrimination, difficulties navigating institutional and financial resources, and loss of social status [2]. For individuals who settled in Australia through Humanitarian Support Programmes, their stress levels are significantly heightened if they resettle from regions of civil conflict and violence, facing financial difficulties and stressful environments either enroute or upon arrival [2].

Adding to the existing precarity, studies have found that approximately 34% of people belonging to CaRM communities have also been victims of racism due to their skin colour, ethnicity, or religion [6,7]. Racism is usually expressed as personal racism (racist attitudes and behaviours), interpersonal racism (racist interactions between individuals), and systemic racism (systemic inequalities within a society due to formal policies, practices, and control of resources) [8]. While racism exists in Australian society, many Australians tend to deny or dismiss that the public hold negative feelings and attitudes about people from certain national and ethno-religious backgrounds [6].

In the wake of the COVID-19 pandemic, there have been significantly high rates of racism, both overt and covert, against CaRM communities in Australia [9]. More recently, religious discrimination, especially Islamophobia, has also contributed to an increase in numbers of more severe forms of racial attacks against people with visual differences in their religion in terms of dress; for example, Muslim women wearing a hijab or men wearing a skull cap [8]. There has also been an increase in the number of more overt forms of racial attacks against Jewish and Arab Israelis due to the ongoing religious and ethnic tensions in the Middle East [10]. As such, the Australian federal and state governments have committed to enforce stricter anti-racism policy and actions, including introduction of a national anti-racism framework and state anti-racism taskforces as a requirement of the Australian Human Rights Commission [11].

### 1.1. New Racism in Healthcare Systems and Impacts on the Mental Health of CaRM Communities

There is now increased awareness that people belonging to CaRM communities are faced with more subtle, insidious, and covert forms of prejudice and discrimination known as “new racism” [12], such as having a deficit-focused, rather than a holistic, view of CaRM people and their psychosocial capabilities to cope with mental health issues [2]. New racism is a term used in modern multicultural societies to refer to a culture-based discrimination of migrants who differ from the prescribed standard [2]. For example, the non-recognition of overseas qualifications from some countries, or the need to take registration exams and experiences from non-English-speaking backgrounds, being judged as having poor English language proficiency based on first or last name and accent, being employed in roles that do not match their qualifications [9], and receiving lower salaries compared to others in the same job are significant forms of prejudice and discrimination that many members of CaRM communities encounter [13]. Individuals faced with new racism may have a higher risk of experiencing poorer mental health, with current findings showing instances of linguistic stigma [4], foreign language anxiety, inferiority complex, insomnia, self-alienation [14], social isolation, and, in more severe cases, depression and suicidal ideations [9,15].

In this study, we draw on the concept of new racism to describe the subtle, culturally coded, and institutionally embedded forms of racialisation experienced by CaRM communities in contemporary mental healthcare. The term was originally introduced by Sniderman et al. (1991) to capture the shift from overt, biologically framed racism to more covert expressions justified through cultural explanations, administrative norms, or ostensibly “race-neutral” discourse [16]. Contemporary scholarship further demonstrates that new racism operates through institutional practices and social narratives that maintain inequity while appearing objective or benign [17,18]. This conceptualisation differs from bias, which reflects individual cognitive attitudes and stereotypes, often implicit [19], and from microaggressions, which refer to interpersonal slights and everyday discriminatory encounters [20]. It also diverges from macro-level racism, which encompasses overt, structural forms of discrimination embedded in policies and systems [21]. We therefore adopt the term new racism because it most accurately captures the subtle, normalised, and institutionally sustained racialisation described by participants, aligning with recent analyses of racism in healthcare systems [22]. A comparative table (Table 1) has been included to clearly delineate these distinctions and situate new racism within the broader landscape of contemporary racism frameworks.

New racism also prevails in healthcare systems [22], with people belonging to CaRM communities experiencing covert racism such as dismissal of health conditions and symptoms, unequal care and treatment, and being left out of the decision-making process [10,23]. These experiences have important implications for mental health nurses, who are often the primary point of therapeutic contact and rely on trust and rapport to deliver effective and culturally responsive care. When individuals feel discriminated against by health professionals, they frequently develop emotions such as frustration, sorrow, dismay, and, in some cases, fear and mistrust of the healthcare system. These responses arise from their lived experiences of racism and can significantly influence how they engage with mental health services [23].

The occurrence of new racism has led to the reluctance of CaRM communities seeking care and treatment, often resulting in presentations only when symptoms have become very severe [24]. When individuals present during the acute phase of their mental illness, mental health nurses face additional challenges, as delayed engagement reduces the time available to build therapeutic relationships, conduct comprehensive assessments, and provide culturally responsive support. This can result in missed opportunities for mental health promotion, illness prevention, and early interventions to improve care and treatment outcomes [25]. Individuals who develop negative feelings such as fear, mistrust, anger, frustration, and dissatisfaction towards health professionals are also less likely to adhere to their medications or care and treatment plans [23], which further affects the continuity and effectiveness of nursing care. Therefore, it is important to address the impacts of new racism on mental health service use among CaRM communities to strengthen culturally safe, equitable, and effective mental health nursing practice.

### 1.2. The Main Aim of This Study

The main aim of this study was to identify factors critical to address new racism in healthcare systems and increase CaRM communities’ use of mental health services. This aim is aligned with the United Nation (UN)’s Sustainable Development Goals (SDG) 3—ensure healthy lives and promote wellbeing for all at all ages, and SDG 10—to reduce inequality within and among countries [26].

### 1.3. Ethical Considerations

This study was part of a Healthway-funded project that received ethics approval from Curtin University Human Research Ethics Committee (Approval number: HRE2024-0576) and Mudoch University Human Research Ethics Committee (Project No 2025/084).

## 2. Methods

### 2.1. Study Design

This study utilises ‘Q’ methodology, which is a ready-to-use mixed method that researchers can adopt without needing to create a new design [27]. Q methodology combines quantitative factor analysis with qualitative interpretation of subjective meaning into one research process [28]. This method enabled the researchers to generate viewpoints that were statistically derived to generate an accurate and in-depth understanding of key factors that CaRM people viewed as critical to address the impact of new racism on CaRM people’s mental health. The Q methodology consisted of five steps: (1) set up of the Q-sorting instrument, (2) selection of participants, (3) data collection, (4) factor analysis, and (5) factor interpretation [28].

### 2.2. Step 1—Set up of the Q-Sorting Instrument

This Q methodology study began with the development of a comprehensive set of statements, known as the concourse [29], representing how CaRM communities understand the factors needed to address the impact of new racism on their mental health. The initial 46 statements in the concourse were drawn from a previous study [2] that conducted eight semi-structured focus groups with participants from ten culturally and linguistically diverse ethnic groups: Bhutanese, Chinese, Indian, Japanese, Filipino, Kenyan, Malawian, Mongolian, Singaporean/Malaysian, and Vietnamese. These statements were then reviewed and refined by the research team using three criteria: (i) fidelity to the perspectives expressed in the earlier study, (ii) clarity and readability, and (iii) conceptual distinctiveness. During this refinement process, the team engaged in reflexive discussion to consider how their professional backgrounds and assumptions about culturally safe mental healthcare might influence decisions about inclusion, exclusion, or wording. No statements were removed arbitrarily; exclusions were made only when items demonstrated conceptual overlap, limited salience, or insufficient relevance to the shared discourse. The resulting 21 statements (Table 2), known as the Q-set [30], were subsequently reviewed by the project’s steering committee, comprising ten CaLD community members representing the groups involved in the focus groups. Finally, the Q-set was uploaded into the online Q Method Software (https://qmethodsoftware.com/, accessed on 2 April 2024) for administration during Step 3 (Data Collection), enabling participants to complete the Q-sort.

### 2.3. Step 2—Selection of Participants

In Step 2 of this Q methodology study, purposive sampling was utilised to recruit people belonging to CaRM communities in Australia to participate in the study. Participants were invited using a recruitment flyer posted in public community settings, on social media, and shared through professional networks. The inclusion criteria were: (i) aged 18 and above, (ii) migrant or having a parent who was a first-generation migrant, and/or (iii) have acted as an interpreter for others with a similar background to oneself in the absence of interpreters. The recruitment flyer contained information about this study, what was expected of the participants if they agreed to participate, and a link to the Q-sorting instrument administered via the online Q Method Software.

### 2.4. Step 3—Data Collection

**Figure 1:**
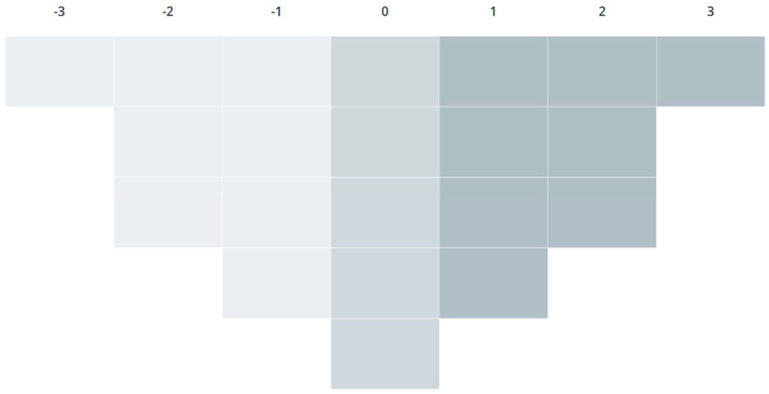
Q-sort grid.

The third step of this Q methodology study involved participants accessing the information sheet, demographic data survey, and instructions on how to complete the Q-sort activity. For instance, participants were instructed to place only one statement card into each box provided in the Q-sort grid (Figure 1).

Participants were also instructed to sort the statements they most disagreed with to the left (−3), the statements that they most agreed with to the right (+3), and the statements that they had a neutral stance towards to the remaining boxes in the centre of the Q-sort grid [28]. The purpose of having the Q-sort grid was to force the participants to sort their responses into a normal distribution to reduce confounding Step 4—Factor analysis [28,31]. However, although the participants sorted the statements into the Q-sort grid, the arrangement of the statements were still based on their own opinion, preserving their viewpoints [32].

### 2.5. Step 4—Factor Analysis

**Figure 2:**
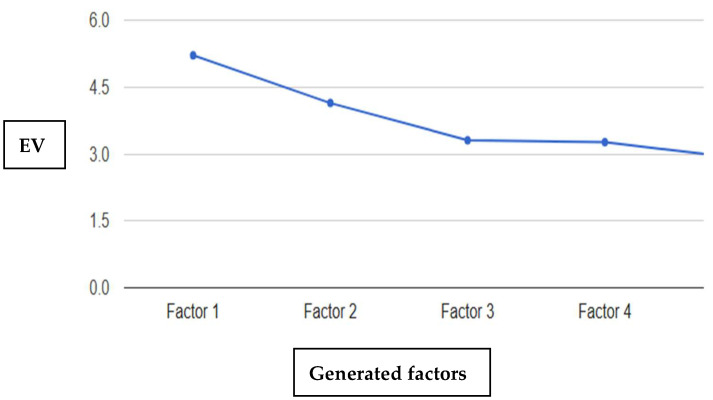
Scree plot test.

Step 4 of this Q methodology study concerned undertaking statistical factor analysis using Principal Component Analysis (PCA) with Varimax rotation function of the Q Method Software to extract unique factors from participants’ Q-sort ratings. This function was used to identify a statistical model that maximises the eigenvalue (EV) for each of the generated factors [26]. After that, the scree plot test (Figure 2) was used to determine the number of generated factors with EVs ≥1.0 to be retained for Step 5—Factor interpretation [28].

According to the scree plot test (Figure 2), factors appearing before the point where the curve forms an ‘elbow’ and the slope begins to flatten should be retained, as they represent the components that explain the greatest proportion of variance [28]. As such, the three generated factors of the graph before the eigenvalues seem to level off were retained for factor interpretation to identify factors critical for addressing new racism in healthcare systems and increasing CaRM communities’ use of mental health services.

### 2.6. Step 5—Factor Interpretation

The final step of this Q methodology study was to interpret the retained three factors and obtain an in-depth and meaningful understanding of participants’ viewpoints [28]. In Q methodology, a factor loading of 0.43 with 21 statements is considered statistically significant at the *p* < 0.05 level, using Equation (1) to calculate the threshold for a significant loading.

(1) $$\frac{1.96}{\sqrt{21}}\approx \frac{1.96}{4.58}\approx 0.43$$

Therefore, distinguishing statements had a statistically calculated Z-score of |Z| ≥ 0.43 of each of the retained factors. Z-scores are calculated to show how the group of participants who share a viewpoint collectively ranked each statement. After that, their Q-sort values, which are the rankings of the statements in the factors, were compared and contrasted with each other to allow meanings to emerge [28]. For instance, statements that the participants shared strongest agreement (+3) or disagreement (−3) with were used for comparisons with the rest of the statements to construct the meaning of the factor and guide the writing of the findings [28].

## 3. Results

Thirty-five people from CaRM backgrounds participated in this study. A total of 27 of the participants identified as female (77.1%) and seven identified themselves as male (20.0%). One of the participants preferred not to say (2.9%). In terms of their age groups, two of the participants (5.7%) were aged between 18 and 19 years, 10 of them (28.6%) were aged between 20 and 29 years, 11 of them (31.4%) were aged 30 to 39 years, six of them (17.1%) were aged 40 to 49 years, and six of them (17.1%) were aged 50 years and above.

Amongst the participants, the majority (62.9%) were Australian citizens, two of them (5.7%) were permanent residents, five of them (14.3%) were on a long-stay visa, and six of them (17.1%) were international students at the time of participation. The majority of the participants (71.4%) had postgraduate qualifications, seven of them (20%) held undergraduate qualifications, and three of them (8.6%) held high school qualifications. In relation to the use of English, 26 of the participants (74.3%) reported that English was a second language, and 23 of them (65.7%) used it at home. The demographic information of the participants is presented in Table 3.

The participants’ personal experiences regarding speaking English, experiences with new racism, and their mental health impacts are presented in Table 4. Although the majority of participants (94.3%) reported feeling confident speaking in English, many of them (68.6%) felt that they were treated differently compared to native English-speaking Australians because of their accent when using the language, skin colour (68.6%), and speaking in another language (62.9%). Notably, most of the participants (74.3%) claimed that they had been in a situation where they felt stressed, angry, sad, or frightened because of others’ lack of understanding of their culture.

Table 5 provides information on the EVs of the three retained factors and the percentage of their cumulative explained variance. Together, the three retained factors accounted for 36% of the cumulative explained variance, which is consistent with expectations in Q methodology where variance is distributed across multiple subjective viewpoints rather than concentrated in a small number of components.

Table 6 provides information on the correlations of the three retained factors. The correlations between factors were small (r = 0.055–0.114), suggesting minimal overlap in the underlying viewpoints. This pattern indicates that the three factors capture meaningfully different perspectives, which aligns with the interpretive aims of Q methodology.

Table 7 presents each participant’s loading on the three retained factors. Participants 6, 11, 12, 20, 22, 26, 27, 28, and 30 loaded significantly onto Factor 1; participants 10, 14, 16, 17, 18, 19, 21, and 29 loaded significantly onto Factor 2; and participants 5, 8, 15, 31, 33, and 34 loaded significantly onto Factor 3 (*p* < 0.05). Participants who did not load significantly onto any factor are those whose Q-sorts did not align strongly with the shared viewpoints represented in the three factors.

### 3.1. Factor 1: Raise Greater Awareness of Mental Health Issues in CaRM Communities (Community-Focused)

Factor 1 was made up of nine participants’ Q-sorts. The age of this group of participants ranged from 30 to 50 and above. Amongst them, eight identified themselves as women (88.9%), seven stated that they have postgraduate qualifications (77.8%), and seven stated that they were Australian citizens (77.7%). The distinguishing statements for Factor 1 are presented in Table 8.

Factor 1 is characterised by 12 distinguishing statements that represented the viewpoints of participants who prioritised public education, open conversation, and positive storytelling about mental health, while rejecting structural or service-level changes such as separate services, formal complaint mechanisms, and culture-specific staffing.

On the one hand, the participants strongly endorsed statements such as to “talk more about the importance of having positive mental health” (S1/+2), “educate CaRM communities on the importance of positive mental health” (S6/+1), “encourage conversations about mental health issues in workplaces, schools, and communities” (S2/+2), and “share positive lived experience stories of CaRM communities with mental illness.” (S9/+1). They also value “promoting awareness of what safe, respectful and inclusive services are” (S3/+3). More significantly, the participants’ disagreement with Statement 13—“stop comparing CaRM Australians to people from their countries of origin” (−2)—highlighted their perspectives on having culturally specific interventions for raising greater awareness of mental health issues in CaRM communities.

On the other hand, they clearly rejected statements that involved major service-focused changes, including to “provide services that are separate from mainstream healthcare services” (S4/−3), “a feedback or complaint mechanism for people who experience new racism when using healthcare services” (S17/−2), a “culture-specific leader, peer support worker, and health professionals” (S7/−1; S8/−1), and “CaRM-friendly policies” (S14/−1).

### 3.2. Factor 2: Provide Visible Anti-Racism and Culturally Safe Services (Service-Focused)

Factor 2 was made up of 10 participants’ Q-sorts. The age of this group of participants ranged from 18 to 39. Amongst them, nine identified themselves as women (90%), seven stated that they have postgraduate qualifications (70%), and six stated that they were Australians (60%). The distinguishing statements for Factor 2 are presented in Table 9.

Factor 2 consisted of 10 distinguishing statements which presented participants who prioritised visible and practical demonstrations of cultural respect within mainstream healthcare settings, while rejecting both the separation of services and community-based awareness initiatives. This factor emphasised the importance of changing the healthcare environment, such as safe spaces for CaRM communities to feel welcomed, safe, and visibly protected from new racism. This interpretation was strongly supported by the participants’ rank-ordered Statement 5: “Make the environment of health services more multicultural; for example, multilingual posters and multicultural decorations” (+3); Statement 18: “Take visible appropriate actions to address new racism in healthcare services” (+2); Statement 21: “Provide culturally appropriate holistic health approaches, wellness, and mental health support for CARM communities” (+2); and Statement 14: “implement CaRM-friendly policies in healthcare services (like being an LGBTQIA+ ally)” (+2). These statements showed a focus on practical, tangible, environmental, and policy cues that signal respect and inclusion.

However, the participants rejected “providing services that are separate from mainstream healthcare services” (S4/−2), highlighting their preference for having safe spaces integrated within mainstream healthcare systems. Additionally, this group of participants differed from participants who contributed to Factor 1 as they rejected statements that emphasise community-focused initiatives, such as “talk more about positive mental health” (S1/−3), “have regular social gatherings to discuss mental health” (S12/−2), or “have multilingual health promotion messages on media” (S11/−1).

### 3.3. Factor 3: Recognise and Formally Address New Racism in Healthcare Systems (Policy-Focused)

Factor 3 was made up of seven participants’ Q-sorts. The age of this group of participants ranged from 20 to 50 and above. Amongst them, six identified themselves as women (85.7%), five stated that they have postgraduate qualifications (71.4%), and three stated that they were Australians (42.9%). The distinguishing statements for Factor 3 are presented in Table 10.

Factor 3 consisted of 11 statements which revealed participants’ view that new racism is a significant problem in mainstream healthcare systems that needs to be openly acknowledged and addressed through strong policy and accountability measures. This interpretation was evidenced by the participants’ rank-ordered Statement 16: “promote awareness of new racism in healthcare services” (+3); Statement 3: “promote awareness of safe and respectful services” (+2); Statement 20: “put clear policies in place that make it possible to address racism through legal means” (+2); Statement 6: “educate CaRM communities on the importance of having positive mental health” (+2); and Statement 10: “respectfully acknowledge and address the cultural and social stigma toward mental health issues” (+1). More importantly, this group of participants’ low rank-ordered Statement 15: “teaching CaRM communities how to talk about mental health” (−2), highlighted their perspective that the CaRM community need to be supported and empowered to express their mental health issues individually.

Nevertheless, these participants’ viewpoints differed strongly from those who contributed to Factor 1 that emphasised the importance of community-focused interventions. This interpretation was supported by their low rank-ordered Statement 11: “have multilingual health promotion messages on social and mainstream media” (−3). Their viewpoint also implied that they would prefer to see higher-level policy and legal safeguards, rather than make administrative and institutional changes like those who contributed to Factor 2. This interpretation was supported by their low rank-ordered Statement 9: “share more positive stories of the lived experiences of CaRM communities with mental illness” (−2); Statement 14: “implement CaRM-friendly policies in healthcare services (like being an LGBTQIA+ ally) (−2); and Statement 17: “provide a feedback or complaint mechanism for people who experience new racism when using healthcare services” (−1).

## 4. Discussion

The three identified factors generated using Q methodology in this study were exploratory and reflect the distinct viewpoints held by participants regarding the conditions they perceived as important for addressing new racism in healthcare systems and increasing CaRM communities’ use of mental health services. The findings do not claim causal relationships but collectively illustrate how participants understood the interplay between community-level, service-level, and policy-level influences on their experiences. These insights may be useful to mental health nurses, who work across these levels and play a central role in shaping culturally safe and equitable care.

Factor 1 presented the viewpoint of participants who felt strongly about increasing general awareness and mental health education within CaRM communities. This finding is aligned with previous research that identified poorer mental health literacy, negative attitudes toward professional help-seeking, shame, preferences for self-reliance, fear of stigma, and concerns about confidentiality as key contributors to the underutilisation of mental health services in CaRM communities [32]. This finding also resonated with the findings of previous studies [33,34] that found that there was a lack of culturally sensitive and responsive assistance that CaRM communities could access to seek help for their mental health. For example, existing health services were neither available in their own language nor did they have staff that could understand the diverse psychosocial or historical factors that contributed to their poor mental health [33,34].

However, it is important to note that strategies that place the onus on CaRM communities to independently access mental health services risk reinforcing stereotyping and implicitly blaming individuals from these backgrounds for experiencing poor mental health [35]. They also minimise the responsibility of healthcare systems to address emerging forms of new racism within healthcare settings and to implement systemic changes necessary to improve mental health service utilisation among CaRM communities [35,36]. As such, there is still a need for healthcare systems to systematically address service-level (Factor 2) and policy-level (Factor 3) issues to holistically address structural and cultural barriers that could limit CaRM communities’ access to mental health services.

Factor 2 presents the viewpoint of participants who placed the responsibility on healthcare systems to address the issue of new racism influencing CaRM communities’ experiences, resulting in barriers to service utilisation. Specifically, this group of participants stressed the importance of healthcare systems demonstrating clear strategies to address racism, cultural respect, and visible inclusion in mental health services. This finding is consistent with that of previous research that highlighted multiple factors underlying the underutilisation of mainstream healthcare systems among CaRM communities, including the lack of culturally sensitive and responsive mental health services, experiences of stigma and discrimination, language barriers, and limited knowledge of available services [37,38].

Having safe spaces integrated within mainstream healthcare systems was an interesting strategy highlighted by the participants as significant to address new racism and increase CaRM communities’ use of mental health services. Safe spaces, in the context of addressing stigma and discrimination within CaRM communities, refer to linguistically inclusive spaces where individuals can feel safe to share lived experiences, emotional struggles, and personal narratives [2,39]. For example, a department within mainstream healthcare systems led by people with lived experiences for CaRM individuals to go to feel safe, listened to, and share or vent negative lived experiences, such as experiencing new racism, with another individual who has faced, endured, and overcome the same experiences [2,40]. More importantly, these safe spaces should be codesigned with CaRM communities to reflect cultural strengths, migration experiences, and collective healing traditions rather than imposing Western clinical framings within mainstream healthcare systems. Additionally, it is important for CaRM communities to know that their negative lived experiences with accessing mental health services need to be reported and followed through appropriate channels within healthcare systems [40].

Interestingly, the provision of more education for staff was not identified as a service-level strategy in this study. While most healthcare systems tend to focus their resources on educating health professionals, the findings of this study resonated with that of Hassen et al., that highlighted the overemphasis on individual-level education as a pitfall in anti-racism interventions [41]. Ricks et al. highlighted that anti-racism and cultural competency education were unable to specifically or significantly address implicit bias, such as the beliefs, attitudes, and behaviours that some healthcare professionals have towards CaRM communities [42]. As such, healthcare systems should re-focus their resources to change practices and policies that seek to dismantle the occurrence of new racism through a multi-level approach [41].

Factor 3 highlighted the viewpoint of participants who advocated for clear recognition, strong policies, and formal accountability to counter racism within healthcare services by naming, recognising, and formally addressing new racism in healthcare. The findings are aligned with that of existing literature [41,42,43] that call for more explicit policies, regulations, and processes which include frameworks, policies, guidelines, and recommendations to address discrimination and racism against CaRM communities.

In most healthcare systems that treat whiteness as the normative reference point from which all others deviate, colour-blindness—for example, being indifferent toward culture and language diversities—could led to policies that perpetuate community deficit and inequitable outcomes for racialised groups [43]. When race and culture are rendered invisible, inequities are misattributed to individual or community shortcomings rather than systemic failure, reinforcing the deficit discourse characteristic of contemporary forms of racism in healthcare. Accordingly, anti-racism policy development must be consumer-driven and co-designed with CaRM communities, ensuring that policy responses directly confront and dismantle these deficit narratives, rather than reproduce them [43].

## 5. Strengths and Limitations of This Study

This study is the first to apply Q methodology to explore how CaRM communities understand the factors needed to address new racism in healthcare and improve mental health service use. The use of Q methodology enabled the identification of statistically derived shared viewpoints, offering an interpretive framework for understanding how different groups prioritise and make sense of these issues. The findings are exploratory rather than generalisable [40], yet the three emergent viewpoints provide a useful conceptual foundation for informing service redesign, policy development, and future research across diverse settings.

Several limitations should be acknowledged. First, recruitment occurred entirely online, meaning the researchers had no control over who chose to participate. This resulted in a self-selected sample whose demographic and experiential characteristics, such as the high proportion of participants with postgraduate qualifications, may not fully represent the breadth of CaRM communities. While this limits demographic representativeness, Q methodology does not aim to estimate population prevalence; instead, it seeks to capture the range of subjective viewpoints within a discourse. The interpretive strength of the findings therefore lies in the coherence of the viewpoints rather than the demographic distribution of participants.

Secondly, CaRM communities are internally diverse, encompassing multiple ethnicities, migration histories, languages, and experiences of racism. Although the concourse was developed from a prior qualitative study involving ten distinct ethnic groups, and the Q-set was reviewed by a CaLD steering committee, the sample cannot capture the full cultural heterogeneity of CaRM populations. The viewpoints identified represent patterned subjectivities among those who participated, not the full spectrum of perspectives that may exist across all CaRM communities.

Thirdly, the study was conducted within the Australian context, where racialisation processes, migration pathways, and health system structures may differ from those in other countries. As such, the viewpoints should be interpreted with sensitivity to the sociopolitical environment in which they were generated.

Finally, online recruitment may have favoured individuals with higher digital literacy, English proficiency, and comfort engaging with research in virtual formats. These characteristics may shape how participants interpret and prioritise issues related to racism and mental health. Future research could complement these findings through community-based, in-person recruitment strategies that engage participants with varied educational backgrounds, migration experiences, and levels of digital access.

Despite these limitations, the study offers a rigorous and culturally grounded exploration of how CaRM communities conceptualise the changes needed to address new racism in healthcare. The use of Q methodology provides a nuanced understanding of patterned subjectivities that can inform future research, policy, and practice.

## 6. Implications for Policy and Practice

This study is the first to use Q methodology to identify statistically derived viewpoints on the conditions participants perceive as important for addressing new racism and improving CaRM communities’ engagement with mental health services in Australia. While the findings are not intended to be generalisable, the three factors offer insight into how culturally and racially marginalised communities conceptualise the barriers and enablers shaping their mental health service experiences. These viewpoints may have implications for policy development, professional practice, and organisational reform, including areas directly relevant to mental health nursing practice.

Collectively, the three statistically derived viewpoints illustrate that participants view new racism as operating across community, service, and policy levels rather than solely within interpersonal interactions. Accordingly, efforts to improve CaRM communities’ experiences of mental healthcare may benefit from approaches that consider structural and organisational influences alongside individual-level strategies. For mental health nurses, this highlights the importance of understanding how racism manifests across multiple levels of the system and how these dynamics shape engagement, trust, and therapeutic relationships. Participants’ viewpoints also potentially point to the need for stronger accountability mechanisms to ensure that commitments to anti-racism translate into sustained change. This may include transparent reporting pathways for racism-related incidents, equity-focused performance indicators, routine monitoring of service outcomes for CaRM communities, and independent oversight of complaints and follow-up actions. Participants further emphasised the importance of co-designed governance structures involving CaRM communities to ensure that accountability processes are culturally grounded, trusted, and responsive.

For policymakers, the statistically derived viewpoints may be used to inform the development of anti-racist healthcare policies, culturally responsive service models, and regulatory frameworks that explicitly address structural contributors to new racism. For practitioners, the findings highlight the importance of building trust, improving communication, and strengthening culturally safe environments that may support help-seeking. Taken together, the statistically derived viewpoints suggest that addressing the impacts of new racism may require a shift from individualised or community-focused interventions toward multi-level, system-oriented approaches that recognise racism as a structural determinant of mental health and healthcare access. This shift has direct implications for mental health nursing practice, education, and leadership, as nurses play a critical role in advocating for equitable systems, challenging discriminatory practices, and delivering culturally safe care.

## 7. Conclusions

The findings of this exploratory study suggest that participants perceive a need for healthcare systems to move beyond symbolic commitments and to embed cultural safety and anti-racism more substantively within mental health service structures. Participants’ viewpoints also emphasised the importance of incorporating mechanisms for accountability, transparency, and meaningful community participation into organisational and policy processes. While not intended to be generalisable, the study offers new insight into how CaRM communities understand the conditions they consider important for addressing new racism and improving engagement with mental health services. These insights are particularly relevant to mental health nursing, as nurses play a central role in fostering culturally safe therapeutic relationships, advocating for equitable service environments, and supporting early and sustained engagement with care. By foregrounding the lived experiences and priorities of CaRM communities, this study contributes to a growing evidence base that can inform future research, guide policy development, and support ongoing efforts to strengthen the cultural responsiveness and equity of mental health systems.

## Figures and Tables

**Table 1:** Conceptual Distinctions Between New Racism, Bias, Microaggressions, and Macroaggressions.

Concept	Definition	Key Features	How It Differs from New Racism
New Racism	A contemporary form of racism that is subtle, covert, normalised, and embedded in institutional practices, cultural norms, and everyday interactions.	Indirect, ambiguous, often denied; framed as “cultural difference,” “individual responsibility,” or “neutral policy”.	New racism is systemic and ideological, not just interpersonal. It masks racial exclusion behind seemingly race-neutral language and practices.
Bias (Implicit or Explicit)	Attitudes, stereotypes, or preferences that influence understanding, actions, and decisions. Can be conscious or unconscious.	Cognitive; may or may not manifest behaviourally; can be positive or negative.	Bias is an individual psychological process, whereas new racism is structural and cultural, shaping norms and institutional responses.
Microaggressions	Everyday verbal, behavioural, or environmental slights that communicate hostile, derogatory, or negative messages to marginalised groups.	Interpersonal; often unintentional; cumulative harm; subtle but identifiable acts.	Microaggressions are specific interpersonal events. New racism is broader, encompassing institutional norms and discourses that legitimise these events.
Macroaggressions	Large-scale, overt, systemic acts of discrimination or exclusion targeting racialised groups.	Structural; visible; includes policies, laws, or institutional practices that disadvantage groups.	Macroaggressions are overt and structural. New racism is covert and normalised, operating through subtle mechanisms rather than explicit exclusion.

**Table 2:** Q-set.

To Address New Racism in Healthcare Systems, and to Increase CaRM Communities’ Use of Mental Health Services, We Need to:
Talk more about the importance of having positive mental health. Encourage conversations about mental health issues in the workplace, at school, and in local community centres. Promote awareness on what safe, respectful, and inclusive services are. Provide services that are separated from mainstream healthcare services. Make the environment of health services more multicultural; for example, multilingual posters and multicultural decorations. Educate CaRM communities on the importance of having positive mental health. Have a culture-specific leader or peer support worker present. Employ more culture-specific health professionals. Share more positive stories of the lived experiences of CaRM people with mental illness. Respectfully acknowledge and address the cultural and social stigma toward mental health issues. Have multilingual health promotion messages on social and mainstream media. Have regular social gatherings/meetings to talk about the mental health of CaRM communities. Stop comparing CaRM Australians/residents to people from their country of origin. Implement CaRM-friendly policies in healthcare services (like being an LGBTQIA+ ally). Teach CaRM communities how to talk about their mental health issues. Promote awareness of new racism towards CaRM communities in healthcare services. Provide a feedback or complaint mechanism for people who experienced new racism when using healthcare services. Take visible appropriate actions to address new racism in healthcare services. Support research initiatives that explore and prevent racism among multicultural groups. Put clear policies in place that make it possible to address racism through legal means. Provide culturally appropriate holistic health approaches, wellness, and mental health support for CaRM communities.

**Table 3:** Participants’ demographic data.

Demographic Data	N = 35 (%)
Age group:	
18 to 19	2 (5.7)
20 to 29	10 (28.6)
30 to 39	11 (31.4)
40 to 49	6 (17.1)
50 and above	6 (17.1)
Gender:	
Men	7 (20.0)
Women	27 (77.1)
Prefer not to say	1 (2.9)
Highest attained education level:	
High school	3 (8.6)
Undergraduate (bachelor, associate bachelor)	7 (20.0)
Postgraduate (graduate certificate, graduate diploma, Masters, PhD)	25 (71.4)
Years living in Australia:	
1–3	6 (17.1)
4–6	7 (20.0)
7–9	3 (8.6)
More than 10 years	19 (54.3)
Residency status:	
Citizen	22 (62.9)
Permanent resident	2 (5.7)
Long-stay visa	5 (14.3)
International student visa	6 (17.1)
English as second language:	
Yes	26 (74.3)
No	9 (25.7)
Speak English at home:	
Yes	23 (65.7)
No	12 (34.3)

**Table 4:** Personal experiences with speaking English, experiences with new racism, and their mental health impacts.

Questions	N = 35 (%)
Do you feel confident speaking in English?	
Yes	33 (94.3)
No	2 (5.7)
Have you ever felt that you were treated differently because of your English?	
Yes	18 (51.4)
No	17 (48.6)
Have you ever felt that you were treated differently because of your accent when speaking in English?	
Yes	24 (68.6)
No	11 (31.4)
Have you ever felt that you were treated differently because of your skin colour?	
Yes	24 (68.6)
No	11 (31.4)
Have you ever felt that you were treated differently because you speak another language?	
Yes	22 (62.9)
No	13 (37.1)
Have you been in a situation where you felt hesitant, stressed, anxious, angry, sad, or frightened because you were speaking in English?	
Yes	18 (51.4)
No	17 (48.6)
Have you been in a situation where you felt stressed angry, sad, or frightened because you were speaking in your first language?	
Yes	10 (28.6)
No	25 (71.4)
Have you been in a situation where you felt stressed, angry, sad, or frightened because of others’ lack of understanding of your culture?	
Yes	26 (74.3)
No	9 (25.7)
Have you ever sought help to support or improve your mental health and wellbeing?	
Yes	13 (37.1)
No	22 (62.9)

**Table 5:** EVs of the three retained factors, the percentage of their explained variance, and the percentage of their cumulative explained variance.

	Factor 1	Factor 2	Factor 3
Eigenvalues	5.21327	4.14072	3.31008
% Explained Variance	15	12	9
Cumulative % Explained Variance	15	27	36

**Table 6:** Score correlations of the factors.

	Factor 1	Factor 2	Factor 3
Factor 1	1	0.09733	0.11419
Factor 2	0.09733	1	0.05496
Factor 3	0.11419	0.05496	1

**Table 7:** Participants’ loading on the three retained factors.

Participant Number	Factor 1	Factor 2	Factor 3
1	0.124	0.0555	0.28299
2	0.06707	−0.47739	−0.00147
3	0.13387	0.49033	−0.06845
4	0.42202	−0.48447	0.2788
5	0.11069	−0.05346	−0.81074 *
6	0.57316 *	0.03629	0.1768
7	0.34746	0.02269	−0.02677
8	−0.15364	0.12688	0.43923 *
9	−0.30877	0.08133	−0.06253
10	0.13365	0.70046 *	0.28622
11	0.79546 *	−0.13129	−0.04345
12	0.52873 *	0.32129	−0.23141
13	−0.03891	0.0452	0.6597 *
14	0.03727	0.43485 *	−0.0766
15	0.0849	0.2144	−0.44247 *
16	0.32378	−0.48581 *	0.06649
17	−0.00148	0.62447 *	0.01739
18	−0.2404	0.55631 *	−0.3444
19	0.17827	0.67607 *	0.15341
20	0.5472 *	−0.14955	0.05369
21	0.49549	0.58219 *	0.01881
22	−0.43162 *	−0.06213	0.17001
23	−0.058	0.22476	0.06252
24	−0.0031	−0.38536	−0.29615
25	0.30309	−0.16444	−0.35432
26	0.64858 *	0.30999	0.2465
27	0.62965 *	−0.00747	0.09747
28	0.46534 *	−0.16569	−0.0572
29	−0.25534	0.54969 *	0.0383
30	0.6943 *	0.19993	−0.20088
31	0.53509	−0.03928	0.62244 *
32	0.40346	0.26032	−0.36516
33	0.18742	0.05862	0.5522 *
34	0.42393	−0.15586	0.53557 *
35	0.25376	−0.10589	0.10484

* Indicates that the participant’s Q-sort is significantly loaded onto the factor.

**Table 8:** Distinguishing Statements for Factor 1.

Statement Number	Statement	Q-Sort Value	Z-Score Value
4	Provide services that are separated from mainstream healthcare services.	−3	−1.83
17	Provide a feedback or complaint mechanism for people who experience new racism when using healthcare services.	−2	−1.31
7	Have a culture-specific leader or peer support worker present.	−2	−1.07
13	Stop comparing CaRM Australians/residents to people from their country of origin.	−2	−0.98
14	Implement CaRM-friendly policies in healthcare services (like being an LGBTQIA+ ally).	−1	−0.97
8	Employ more culture-specific health professionals.	−1	−0.63
6	Educate CaRM communities on the importance of having positive mental health.	+1	0.96
9	Share more positive stories of the lived experiences of CaRM people with mental illness.	+1	1.02
2	Encourage conversations about mental health issues in the workplace, at school, and in local community centres.	+2	1.27
10	Respectfully acknowledge and address the cultural and social stigma toward mental health issues.	+2	1.35
1	Talk more about the importance of having positive mental health.	+2	1.49
3	Promote awareness of what safe, respectful, and inclusive services are.	+3	1.60

**Table 9:** Distinguishing Statements for Factor 2.

Statement Number	Statement	Q-Sort Value	Z-Score Value
1	Talk more about the importance of having positive mental health.	−3	−1.76
4	Provide services that are separate from mainstream healthcare services.	−2	−1.69
13	Stop comparing CaRM Australians/residents to people from their country of origin.	−2	−1.27
12	Have regular social gatherings/meetings to talk about the mental health of CaRM communities.	−2	−0.96
7	Have a culture-specific leader or peer support worker present.	−1	−0.78
11	Have multilingual health promotion messages on social and mainstream media.	−1	−0.72
21	Provide culturally appropriate holistic health approaches, wellness, and mental health support for CaRM communities.	+2	1.00
14	Implement CaRM-friendly policies in healthcare services (like being an LGBTQIA+ ally).	+2	1.19
18	Take visible appropriate actions to address new racism in healthcare services.	+2	1.85
5	Make the environment of health services more multicultural; for example, multilingual posters and multicultural decorations.	+3	1.89

**Table 10:** Distinguishing Statements for Factor 3.

Statement Number	Statement	Q-Sort Value	Z-Score Value
11	Have multilingual health promotion messages on social and mainstream media.	−3	−1.90
15	Teach CaRM communities how to talk about their mental health issues.	−2	−1.50
9	Share more positive stories of the lived experiences of CaRM communities with mental illness.	−2	−1.13
14	Implement CaRM-friendly policies in healthcare services (like being an LGBTQIA+ ally).	−2	−0.82
17	Provide a feedback or complaint mechanism for people who experience new racism when using healthcare services.	−1	−0.82
19	Support research initiatives that explore and prevent racism among multicultural groups.	−1	−0.78
10	Respectfully acknowledge and address the cultural and social stigma toward mental health issues.	+1	0.80
3	Promote awareness of what safe, respectful, and inclusive services are.	+2	1.20
20	Put clear policies in place that make it possible to address racism through legal means.	+2	1.21
6	Educate CaRM communities on the importance of having positive mental health.	+2	1.21
16	Promote awareness of new racism towards CaRM communities in healthcare services.	+3	2.15

## Data Availability

Data collected in the study are not publicly available due to privacy and ethical restrictions but can be made available upon reasonable request from the corresponding author.

## Public Involvement Statement

CaRM people in Australia were involved in this research through participating in the focus groups.

## Guidelines and Standards Statement

This study was reported in accordance with the Consolidated Criteria for Reporting Qualitative Research (COREQ), with adaptations appropriate for Q methodology.

## Abbreviations

The following abbreviations are used in this manuscript:

CaLD	Culturally and Linguistically Diverse
CaRM	Culturally and Racially Marginalised
COVID	Coronavirus disease
